# Discrete Molecular Dynamics Can Predict Helical Prestructured Motifs in Disordered Proteins

**DOI:** 10.1371/journal.pone.0095795

**Published:** 2014-04-24

**Authors:** Dániel Szöllősi, Tamás Horváth, Kyou-Hoon Han, Nikolay V. Dokholyan, Péter Tompa, Lajos Kalmár, Tamás Hegedűs

**Affiliations:** 1 MTA-SE Molecular Biophysics Research Group, Hungarian Academy of Sciences, Budapest, Hungary; 2 Department of Biophysics and Radiation Biology, Semmelweis University, Budapest, Hungary; 3 Institute of Enzymology, Research Centre for Natural Sciences, Hungarian Academy of Sciences, Budapest, Hungary; 4 Department of Bioinformatics, University of Science and Technology, Yuseong-gu, Daejeon, Korea; 5 Biomedical Translational Research Center, Division of Convergent Biomedical Research, Korea Research Institute of Bioscience and Biotechnology, Yuseong-gu, Daejeon, Korea; 6 Department of Biochemistry and Biophysics, UNC at Chapel Hill, Chapel Hill, North Carolina, United States of America; 7 VIB Department of Structural Biology, Vrije Universiteit Brussel, Brussels, Belgium; 8 Institute of Molecular Pharmacology, Research Centre for Natural Sciences, Hungarian Academy of Sciences, Budapest, Hungary; Weizmann Institute of Science, Israel

## Abstract

Intrinsically disordered proteins (IDPs) lack a stable tertiary structure, but their short binding regions termed Pre-Structured Motifs (PreSMo) can form transient secondary structure elements in solution. Although disordered proteins are crucial in many biological processes and designing strategies to modulate their function is highly important, both experimental and computational tools to describe their conformational ensembles and the initial steps of folding are sparse. Here we report that discrete molecular dynamics (DMD) simulations combined with replica exchange (RX) method efficiently samples the conformational space and detects regions populating α-helical conformational states in disordered protein regions. While the available computational methods predict secondary structural propensities in IDPs based on the observation of protein-protein interactions, our *ab initio* method rests on physical principles of protein folding and dynamics. We show that RX-DMD predicts α-PreSMos with high confidence confirmed by comparison to experimental NMR data. Moreover, the method also can dissect α-PreSMos in close vicinity to each other and indicate helix stability. Importantly, simulations with disordered regions forming helices in X-ray structures of complexes indicate that a preformed helix is frequently the binding element itself, while in other cases it may have a role in initiating the binding process. Our results indicate that RX-DMD provides a breakthrough in the structural and dynamical characterization of disordered proteins by generating the structural ensembles of IDPs even when experimental data are not available.

## Introduction

In the last decade it became evident that a significant portion of proteins in every organism exhibits disordered regions without stable secondary or tertiary structures [1], [2]. This notion has changed the structure-function paradigm and led to the re-assessment of basic notions of structural biology by suggesting that tertiary structure is not the prerequisite of protein function. Intrinsically disordered proteins and protein regions function in various important cellular processes, such as transcription regulation, mRNA processing, differentiation, and apoptosis. Their molecular mechanisms often involve protein-protein interactions, in which the structural flexibility of IDPs enables a high specificity associated with low affinity. Their binding to the interaction partner frequently proceeds via induced folding and is in the focus of attention because it is also frequently involved in pathological conditions (e.g. Parkinsons disease, Alzheimer's disease, cancer) [3], [4]. The special mode of binding also serves as a basis of drug design for more effective treatments [5], which can be facilitated also by knowledge on the conformational ensemble of the unbound IDP. However, most of the current experimental and computational tools are developed for proteins with stable structures. Importantly, it has become evident that IDPs are not completely disordered and may exhibit transient short and long range structural organization related to function. NMR methods have demonstrated transient secondary structural elements in disordered regions [6], which are often involved in binding and are thus termed MoRFs (Molecular Recognition Features), MoREs (Molecular Recognition Elements), PSEs (Preformed Structural Elements) or PreSMos (Pre-Structured Motifs) [6]. Since experimental approaches to characterize disordered proteins and their complexes are limited in many ways (e.g. by labeling, expression and purification of IDPs, size limitations in high performance NMR, and simple time and resource constraints of experiments for thousands of IDPs in different proteomes), there is a demand for computational methods to characterize the conformational space of IDPs.

The algorithm Flexible-meccano has been developed to sample the entire conformational space available for IDPs, based on amino acid-specific conformational potentials and volume exclusion [7]. Restrained molecular dynamics simulations are also used for describing conformational ensembles, usually employing distance constraints derived from NMR, as in the case of α-synuclein [8]. However, *ab initio* methods so far to generate and characterize ensembles of IDPs encompass serious limitations [9], [10]. Conventional molecular dynamics simulations are difficult to employ for the representative sampling of the entire conformational space because of extreme conformational freedom and astronomical numbers of possible conformations.

Discrete Molecular Dynamics (DMD) may offer an alternative because it provides a higher performance and better sampling compared to conventional MD [11]. The increased performance of DMD derives from its collision driven algorithm, in which the energy of the system is recalculated not at specific time points but only at the time of the next collision. In addition, the solvent is modeled implicitly, which decreases the time needed for energy calculations and accelerates motions in the system. The high accuracy of description of events at the atomic level is assured by its force field based on CHARMM [12]. Long-range electrostatic interactions, which allow modeling of salt-bridges, are also implemented. To further increase sampling, DMD can be combined with replica exchange (RX) [13], in which several replicas are run in parallel, at various temperatures. Temperatures of replicas are exchanged in a Metropolis-based stochastic manner, to ensure that replicas can escape from a trapped state at a higher temperature, which leads to a much increased sampling of the conformational space.

To show that RX-DMD is a valuable method for describing conformations of disordered proteins, we performed simulations of IDPs that contain experimentally characterized α-PreSMos. We correlated our calculations with these NMR-based helical PreSMos [6] and also with X-ray structures, in which the disordered region is folded in the presence of the partner (α-MoRF regions [14]).

## Methods

### Input sequences, structure generation, and energy minimization

Two sets of protein sequences were used to generate extended structures in PyMol (The PyMOL Molecular Graphics System, Version 1.5.0.1 Schrödinger, LLC) for molecular dynamics simulations: (1) 25 sequences of constructs used in NMR experiments and with recognized PreSMos [6]. (2) 323+4 sequences from complexes from PDB, in which the recognition region of a disordered protein exhibits a visible and well-defined secondary structure (P. Tompa, unpublished; plus four membrane proteins selected using mpMoRFsDB [15]). The structures were energy minimized by the DMD [11] protocol of Chiron (http://troll.med.unc.edu/chiron) [16]. Briefly, a short simulation (1,000 time unit) using a high heat exchange factor (HEX = 10) at a high temperature (0.7 temperature unit) was performed followed with a short simulation with a low heat exchange factor (HEX = 0.1) at a low temperature (0.5 temperature unit). Cα and Cβ atoms were restrained. In all DMD simulations, including those combined with replica exchange a united-atom representation is used to model proteins, in which all heavy atoms and polar hydrogen atoms of each amino acid are included [11], [13]. The van der Waals and solvation interactions are pair-wise functions of distances, while the hydrogen bonds are angular- and distance-dependent multi-body interactions. The solvent is implicitly modeled employing the Lazaridis-Karplus solvation model [17]. Long range electrostatic interactions, which allow modeling of salt-bridges, are also implemented [11]. The πDMD software employed for simulations was kindly provided by Molecules in Action, LLC (http://www.moleculesinaction.com). These short simulations completed in 10 minutes on 8–16 processors of our local HPC. Sequences, exact sequence boundaries used in simulations, and configuration files can be found at http://disorder.hegelab.org.

### Replica exchange DMD simulations

RX-DMD simulations [13] were performed with 8 replicas at temperatures 0.5246, 0.5451, 0.5665, 0.5886, 0.6116, 0.6355, 0.6604, and 0.6862 temperature unit, for 1,000,000 time units. Most of the disordered peptides were fully elongated at the highest temperatures. Conditions for replica exchange were tested every 1,000 time units and frames were saved every 200 time units. This way, 5,000 conformations were generated for each replica. Temperatures were selected to ensure at least 25% exchange probability between two temperatures with polypeptides of 80–100 residues in length. Although full optimization of the temperature range is impossible for the number of proteins investigated in our study, it is not critical because of the flat potential energy surface of disordered proteins. Anderson's thermostat was used and the heat exchange factor was set to 0.1. At the end of a simulation, the frames (conformations) from every trajectory were grouped by temperature for analysis. These simulations run on the Hungarian HPC infrastructure (NIIF Institute, Hungary) and the HPC of the Institute of Enzymology (RCNS, HAS, Hungary, supported by the Momentum Program of HAS), completed in 3–11 days, depending on the length of the simulated polypeptide. Each run produced 8–20 Gb of raw and analyzed data.

### Secondary structure analysis

Ψ and Φ torsion angles were determined by DSSP [18] for every structure at every temperature. The occurrence of torsion angles characteristic of α-helices was counted for every amino acid position and was divided by the total number of the structures (5,000). Matching of regions with α-helical propensities determined by RX-DMD with the experimental PreSMos was performed manually. Decision of the match was determined by very similar boundaries (+/−2 amino acids) or significant overlap of the peaks, complemented with the observation of the given α-helical regions at different temperatures. Unfortunately, β-PreSMos cannot be detected, since the torsion angles of amino acids in sheets are also characteristic of the elongated conformations of disordered polypeptides. To see if the α-helical torsion angles arise at the level of individual amino acids or continuous helices are formed, the helical propensities for each frame were plotted along the amino acid sequence. Ramachandran plots were also plotted using Ψ and Φ angles determined by DSSP. All calculations and plotting were done in R [19].

### Density of States (DoS) calculation

To determine the distribution of different conformations in an ensemble, the free energy surface of the given protein can be calculated, which is a challenging task. However, the potential surface of a protein can also be described by the distribution of conformations along one or more reaction coordinates, based on the assumption that the simulations create ensembles with a Boltzmann-distribution. This meets the assumption that structures observed at a higher frequency correspond to more favorable, low energy states. To this end, we selected two reaction coordinates (the radius of gyration (R_g_) and the energy (E)), which are commonly used for describing potential surfaces because of their simplicity. This PMF-like function was calculated according to Sippl's well-known equation [20]:
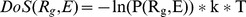
where DoS is the calculated density of states, k is the Boltzmann constant, T is the absolute temperature and P(R_g_,E) is the relative density of the structures with R_g_ gyration radius and E energy. The relative density was determined by calculating the conditional probability of a state with given R_g_ and E. As the chance that two states with the exactly same R_g_ and E values would occur, is almost zero, we counted the structures at a given temperature within a small range (a histogram bin) of R_g_ and E values, and divided it by the total number of structures at the given temperature. R_g_, which describes the packing of a molecule, was calculated as the root mean squared Euclidean distance of the α-carbon atoms from their geometrical center. The energy of each structure was calculated by πDMD during simulation, and was taken from its output. In the case of proteins with a well-defined structure, the smallest R_g_ usually corresponds to the native conformation with a low energy (left-bottom corner of the surface). As an ordered polypeptide, an approximately 100-residue long segment of the MRP1 nucleotide binding domain (PDBID:2CBZ, a.a. 711–821) was selected, because it has a length comparable to that of the investigated proteins.

## Results and Discussion

### α-PreSMo regions detected by RX-DMD highly correlate with data from NMR experiments

We employed a set of 25 proteins with known PreSMos determined by NMR to validate RX-DMD predictions. A fully extended conformation of each disordered segment was generated by PyMol. These structures were energy minimized in two steps while constraining the backbone atoms, using the DMD protocol of Chiron [16]. The input structures were subjected to RX-DMD simulations using 8 replicas (at temperatures: 0.5246, 0.5451, 0.5665, 0.5886, 0.6116, 0.6355, 0.6604, and 0.6862 temperature unit) for 1,000,000 time unit. Although these values correspond to temperatures ranging from −9 to +72°C and to times up to approx. 20 ns, it is important to emphasize that they do not fully match real physical scales because of the collision-based algorithm of DMD and the implicit solvent model [11]. Since the evolution of structures in RX simulations cannot be strictly interpreted in kinetic terms, the structures in each trajectory were grouped based on temperature for analysis.

**Figure 1:**
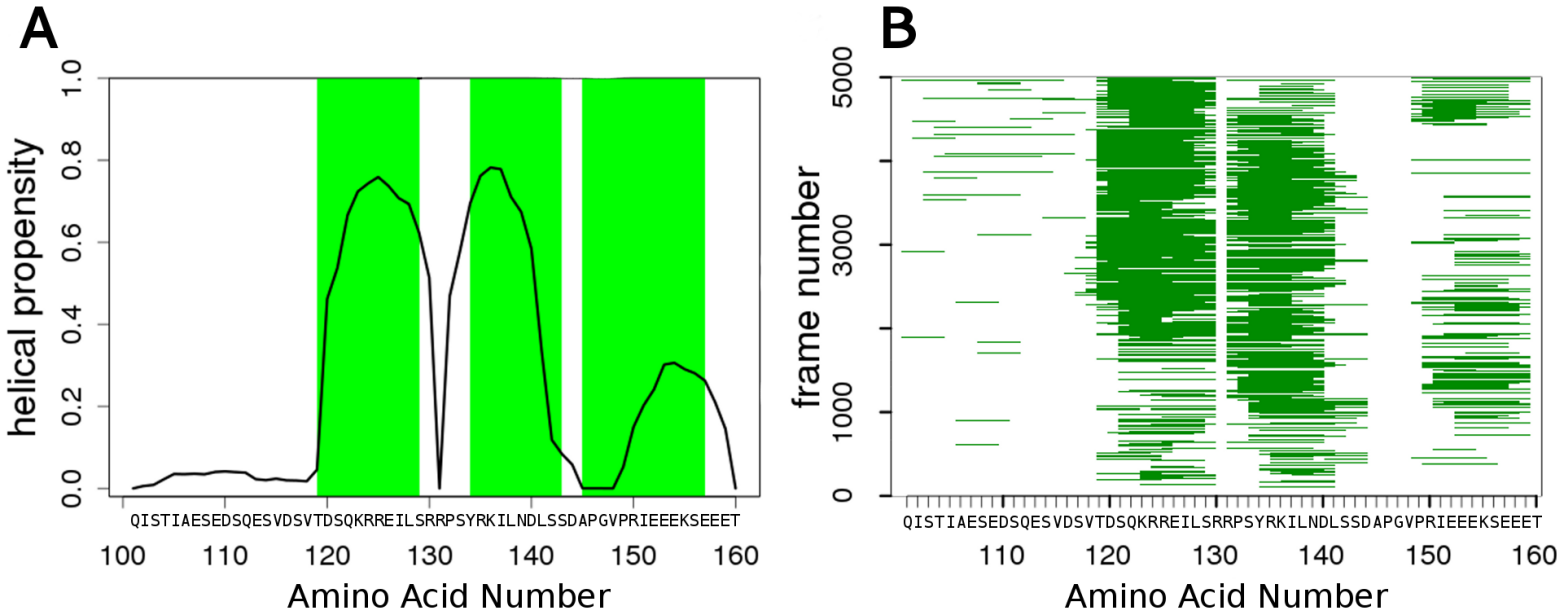
RX-DMD (replica exchange discrete molecular dynamics simulations) predicts prestructured motifs (PreSMos) with high confidence. Secondary structure propensities and a.a. torsion angles were collected using DSSP from conformations of KID (a.a. 101–160) generated by RX-DMD. Regions determined as PreSMos by NMR experiments are labeled with green boxes. (**A**) Probability of amino acids being in a helix conformation in a simulation is shown. Data from 5,000 frames at 0.5886 temperature unit are averaged and normalized. Plots for other temperatures are shown in Figure S1 in File S1. (**B**) Continuous helices observed in various KID conformations are depicted over frames from the same temperature and RX-DMD simulation.

First, secondary structural elements (α-helix and extended strand) were assigned in every 5,000 structures at each temperature using DSSP [18]. The results are illustrated by the average values at a given temperature for CREB KID (Figure 1A). The two α-MoRFs present in the crystal structure of the pKID/CBP complex (PDBID:1KDX; Phosphorylated Kinase Inducible Domain of CREB and KIX domain of CREB Binding Protein), which appear as PreSMos in solution by NMR experiments [21], clearly show up on our RX-DMD simulations. One of them (a.a. 119–129) is populated over 50% at 0.5886 temperature unit (∼23°C) as in the NMR experiments, while the second (a.a. 134–143) is populated similarly, at a level higher than experimentally observed *in vitro*. RX-DMD can also detect a C-terminal α-PreSMo with a lower probability, in contrast to NMR experiments suggesting a preference for β-structures in this region. These differences may be attributed to different structural propensities realized at different temperatures (Figure S1 in File S1). This cannot be tested, since β-PreSMos are difficult to be detected because of the proximity of torsion angles in sheets and other extended conformations, in which disordered polypeptides reside in a significant portion of time. An additional N-terminal α-PreSMo with a very low probability is also predicted for KID by RX-DMD. Comparably good predictions were achieved for all the 24 other experimental PreSMos (Table 1). From 65 PreSMos in these proteins 45 were detected by RX-DMD indicating better performance in predicting secondary structural propensities compared to other methods, such as Agadir [22] (Figure S2 in File S1).

**Table 1:** Prediction of RX-DMD compared to experimental NMR data on PreSMos.

Name (UniProt entry name)	Experimental PreSMos	RX-DMD PreSMos
KID (CREB1_HUMAN)	119–129, 134–143, 145–157	121–130, 132–142, 150–159
APPC (A4_HUMAN)	744–747, 751–759, 761–769	743–758, 761–769
CFTR (CFTR_HUMAN)	654–668, 759–764, 766–776, 801–817	656–672, 677–738, 759–785, 798–815, 826–837
DYIN (DYIN_DROME)	223–228	209–212, 215–219*
ENSA (ENSA_HUMAN)	32–36, 48–50, 65–70	29–40, 51–67, 71–80
ERD14 (ERD14_ARATH)	24–34, 67–77, 90–98, 111–123, 158–165	27–36, 47–57, 64–77, 93–100, 109–125, 158–167, 176–181
FLGM (FLGM_SALTY)	42–50, 60–73, 83–90	41–51, 63–73, 74–95
HBV (Q8JVC8_HBV)	32–36, 41–45, 11–18, 22–25, 37–40, 46–50	79–83*
HCV (Q0MR50_9HEPC)	287–296,325–335	253–266, 292–305
HIV (NEF_HV1BR)	14–22, 35–41	15–23, 32–41
HMGA (HMGA1_HUMAN)	3–9, 64–67	88–96
IPP2 (IPP2_HUMAN)	36–42, 96–106, 127–154	36–55, 96–112, 129–157
LEF1 (LEF1_MOUSE)	9–24, 30–41, 46–66	7–24, 31–41, 42–63, 73–83
P53 (P53_HUMAN)	18–26, 40–44, 48–53	16–24, 47–55
PPR (PPR1B_RAT)	22–29, 103–114	4–8, 25–31, 35–40, 103–114
PTTG (PTTG2_HUMAN)	150–159	16–26, 21–45, 58–64, 112–116, 133–139, 145–151, 155–161, 174–178
RPS4 (RS4_GEOSE)	12–15, 30–33	8–14, 40–61, 67–71, 83–101, 147–157, 191–198
SML (SML1_YEAST)	1–14, 20–35, 61–80	3–10, 58–85, 89–98
SYUA (SYUA_HUMAN)	1–5, 6–37, 38–140	2–11, 20–32, 34–39, 55–64, 75–105, 130–134*
SYUB (SYUB_HUMAN)	1–134	2–35, 48–66, 124–130*
SYUG (SYUG_HUMAN)	49–99	2–9, 19–40, 53–68, 79–85, 116–124
TMOD (Q9DEA6_CHICK)	24–35	4–22, 49–67
VAMP (VAMP2_HUMAN)	10–20, 25–77, 78–91	24–40, 43–72, 78–95*
VP16 (VP16_HHV11)	424–433, 442–446, 465–467, 472–479	436–446, 469–483
WASP (WASP_HUMAN)	252–264	222–235, 241–249, 255–262

*at 0.5246 temperature unit; all other PreSMo regions were defined based on the helical propensity determined at 0.5886 temperature unit.

### Structural properties of RX-DMD conformational ensembles

We also analyzed whether the identified preformed helix regions were the result of individual amino acids transiently sampling characteristic torsion angles with a high frequency, or of continuous helices that formed and persisted through time and replicas. Therefore helical propensities in the KID sequence were plotted for each frame (Figure 1B). The two helices observed in both the crystal structure of the pKID/CBP complex and in solution by NMR prevail in most of the frames in our simulations. Although the helices in the flanking regions appear less frequently, when they exist, they also form continuous helical turns of 6–7 amino acids [23]. The higher probability of the C-terminal flanking α-PreSMo suggests a more likely function of this region in binding compared to the N-terminal segment. It is to be noted that the presence of flanking regions makes KID binding stronger [23], which suggests the role of the terminal sequences in binding and possibly the development of some structural elements in these regions.

**Figure 2:**
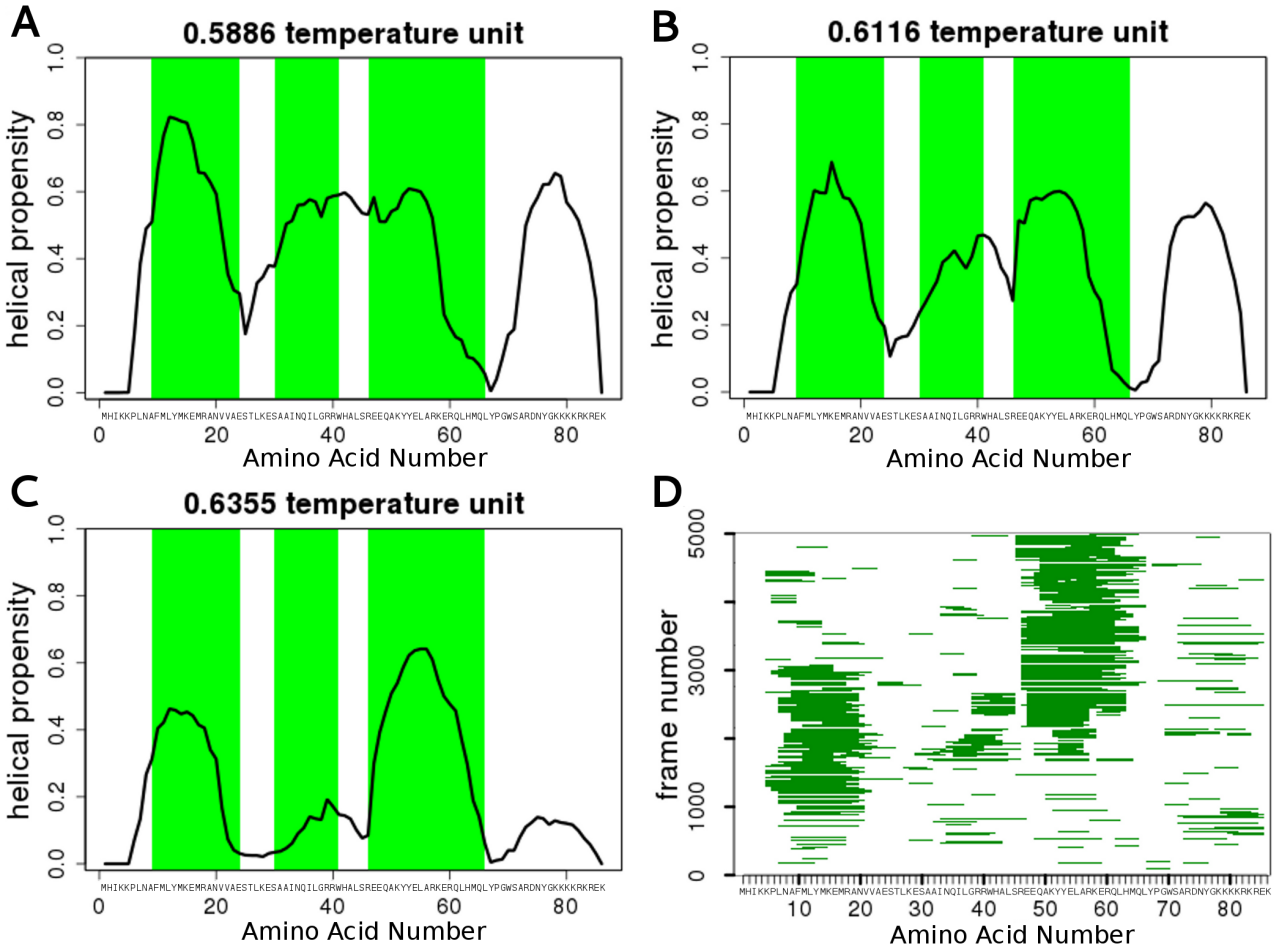
The different temperatures of RX-DMD simulations reveal the details of PreSMo formation and stability. (**A–C**) Secondary structure propensities and a.a. torsion angles were collected and plotted for conformations of LEF1 (a.a. 1–86). Regions determined as PreSMos by NMR experiments are labeled with green boxes. (**D**) Continuous helices observed in various LEF1 conformations are depicted over frames corresponding to conformations at 0.5886 temperature unit.

Our simulations also show that the application of multiple temperatures in RX simulations is highly advantageous, since different PreSMos exhibit different stabilities, thus their propensity varies with the temperature. In accord, performing simulations at different temperatures and analyzing data at all temperatures also provides hints on the stability and experimental detectability of PreSMos and may help in dissecting two closely located PreSMos. These features are illustrated by LEF1. The α-helical propensity plot at 0.5886 temperature unit indicates one long continuous α-PreSMo between a.a. 25 and 60 instead of two (Figure 2A) [24]. At higher temperatures (0.661 and 0.6355 temperature unit) the two helices already appear in the α-helical propensity profiles (Figure 2B,C). Moreover, the propensity of the two α-PreSMos (a.a. 31–41 and 73–83) decreases, indicating that the probability of their folding is smaller. These observations indicate that the different temperatures of RX-DMD simulations are important not just for increasing sampling, but also for proper analysis and conclusions with regards to subtle features. Nevertheless, when plotting the evolution of helical structures over frames, the experimentally determined two α-PreSMos can be identified (Figure 2D) [24]. Importantly, an extreme C-terminal PreSMo, not detected by experiments, is indicated by our simulations. The simulations show that this PreSMo is not so stable (its helical propensity drops at higher temperatures), which may be a reason for its invisibility in experiments. A simple resonance assignment problem in NMR spectra could cause the loss of this information. Although the existence of this C-terminal motif should be tested experimentally, it also suggests that the sensitivity of RX-DMD can serve as input for further experimental design.

**Figure 3:**
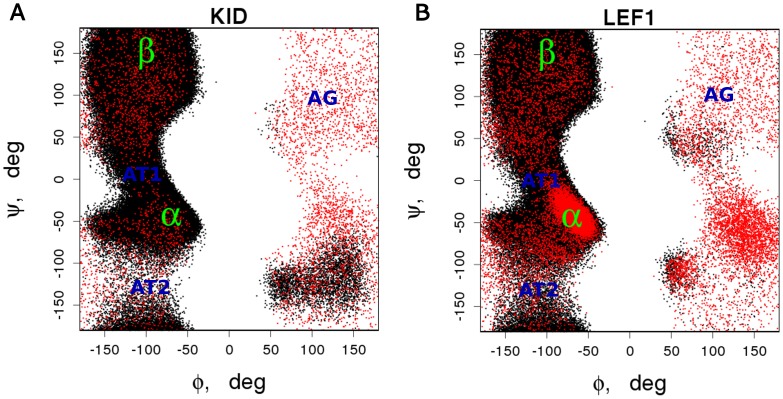
The even distribution of Gly torsion angles may be a characteristic future of disordered proteins. Ramachandran diagrams of (**A**) KID and (**B**) LEF1 conformations plotted using torsion angles determined for every residue in frames at 0.5886 temperature unit, using DSSP.

To further analyze our structural ensembles, we plotted the torsion angles in Ramachandran diagrams (Figure 3). A significant portion of points occupy the areas corresponding to regular helices, and a large set of points are located in the area of extended conformations encompassing β-strands. The transition between extended and helical conformations proceeds most of the time in the area (AT1) around Ψ = 0 and Φ = (−80, −100). The same transition with lower probability is in the area (AT2) Ψ = (−70, −170) and Φ = (−180, −50). Interestingly, there is a non-occupied area between Φ values 50 and −50, and an area (AG) evenly populated with low probability. This AG area is characteristic of angles of flexible glycine. However, in ordered proteins the distribution of the points in this area is discrete indicating that even glycines are restrained due to their incorporation of well-defined structure [25]. Therefore the AG and T2 areas may be characteristic of conformations of disordered proteins and may help in detecting disorder.

Although DMD predicts α-PreSMos in disorder proteins well, it would be important for future studies to determine whether the conformational ensemble simulated correlates well with conformations derived from NMR experiments. However, there is no gold standard method to derive structural ensembles for disordered proteins from NMR studies. In most of the current approaches different computational methods are used to generate a large set of structures, from which a subset is derived that satisfy certain constrains from NMR experiments [7], [8], [26]. Most likely our protocol also provides a set of conformations which only partially correspond to current experiments, which is also suggested by a higher level of α-helical propensities of some PreSMos determined by RX-DMD compared to experimental data (e.g. the second PreSMo in KID). Nevertheless, the distribution of R_g_ values of α-synuclein (an asymmetric peak between 20 and 30 Å; Figure S3 in File S1) at lower temperatures is similar to that coming from NMR experiments done at 4°C [8].

### Predicting conformations of disordered regions in complexes and membrane proteins

To further validate that our method can dissect functional elements in disordered proteins, we also performed simulations on proteins derived from a set of X-ray structures, in which a folded segment of IDPs can be observed. Although this set provides more than 300 observations compared to the 25 cases of NMR experiments in solution, its information content is conceptually different. The structured segments (MoRFs) observed in X-ray complexes may not be pre-formed in solution and their folding may be strongly coupled to binding. In addition, boundaries of PreSMos in solution and MoRFs in complex may or may not be identical. Thus, only a partial overlap of our α-PreSMo prediction and observed experimental MoRFs is expected (Table S1 in File S1). For example, in many cases the predicted regions with α-helical propensities are located next to the structured segment (Table S1 in File S1), i.e. the predicted PreSMo does not correspond to an observed structured segment. However, a PreSMo may initiate the binding and help the formation of the structural element observed in the X-ray structure, thus its knowledge may help in understanding the binding process.

Disordered regions of membrane proteins are also parts of complexes in many times. The sequence-based algorithms [27] to predict PreSMos are influenced by the different sequence composition of disordered segments in the intra- and extracellular parts of transmembrane proteins, compared to that of soluble proteins [27]. Our simulations show that RX-DMD can also predict α-MoRFs/PreSMos in membrane proteins (e.g. CAC, SLC9A1, AMFR) with good performance (Table S1 in File S1).

### Energy surfaces of disordered proteins explored by RX-DMD

**Figure 4:**
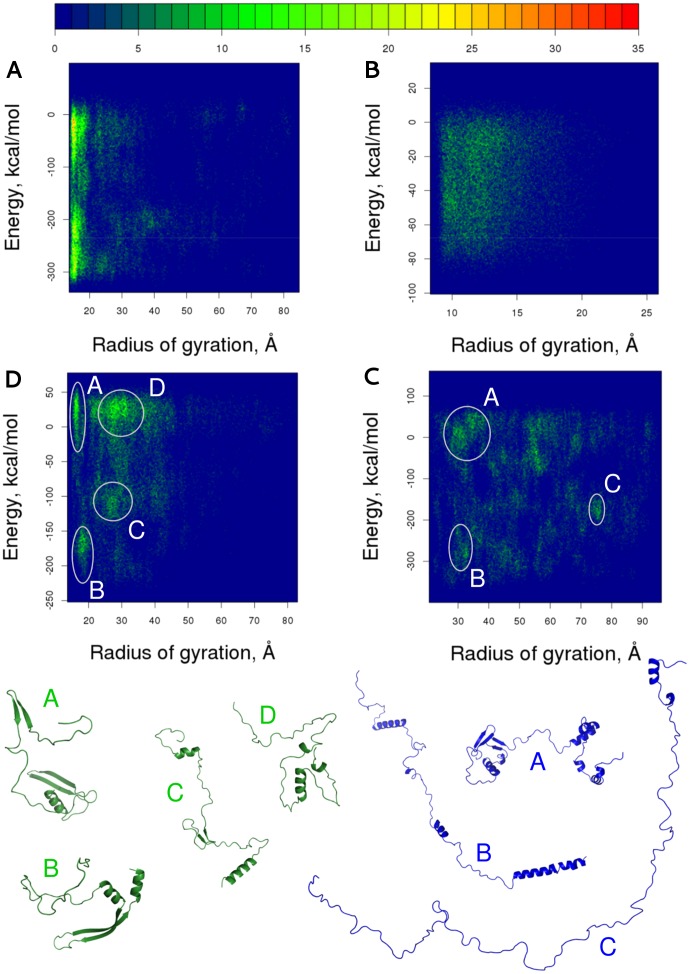
The energy landscape of IDPs is different from that of proteins with stable structures. Density of States (DoS) are calculated along the reaction coordinates R_g_ and energy. (**A**) As an ordered polypeptide, an approximately 100-residue long segment of the MRP1 (a.a. 711–821) was used. (**B–D**) Disordered segments of dynein (a.a. 198–237), protein phosphatase inhibitor (a.a. 9–164), and FlgM (a.a. 1–97). Those conformations of the latter two proteins that were found in and around the minima were clustered based on RMSD. The centroids of the most populated clusters are shown at the bottom (green and blue, respectively). Densities are colored according to the bar at the top.

The success of RX-DMD in predicting α-PreSMos may originate not only from the features of DMD, but also from the properties of potential energy surfaces of disordered proteins, which are expected to contain shallower basins. Their smaller energy barriers allow easier transitions between conformational states that may enable to find characteristic energy minima faster. To characterize the potential surface of IDPs, we calculated Density of States (DoS) along the reaction coordinates R_g_ (radius of gyration) and energy. DoS of the α-helical subdomain of MRP1 nucleotide binding domain, as an example for ordered peptides, exhibits two large clusters with small R_g_ values (Figure 4A). One with higher energies may represent folding intermediates. In general, conformations with large R_g_ values are rare. In contrast, IDPs exhibit different types of energy surfaces. DoS calculated for dynein (DYIN_DROME) shows a flat surface without deep basins. There are also characteristic DoS surfaces for IDPs (e.g. Securin-2, PTTG2_HUMAN; Synaptobrevin-2, VAMP2_HUMAN; Dehydrin ERD14, ERD14_ARATH and Protein phosphatase inhibitor 2, IPP2_HUMAN) which exhibit basins (Figure 4C), although the number of minima is higher and their depth is smaller compared to those of the structured MRP1 NBD1. Interestingly, the DoS of FlgM (O66683_AQUAE) is somewhat similar to that of an ordered protein exhibiting a basin with low energy and R_g_ values (Figure 4D). However, most of its conformations are located on the energy surface area characteristic of structures with high energy and R_g_.

## Summary

DMD not only successfully predicts α-PreSMos without any previous knowledge, but it also provides additional information to predictors of binding regions, because it can detect regions which do not participate directly in binding but exhibit secondary structural propensities. The relatively low computational cost of DMD opens new avenues for describing the dynamics of disordered peptides both in solution and in complex. Compared to other *ab initio* computational studies describing conformational ensembles of small proteins [9], [10], significantly less computational time was needed for RX-DMD simulations with larger proteins (∼64 hours versus ∼1.4 hours). This performance, which cannot be exceled by conventional all-atom simulations even on super-fast computers, allowed us to show for a large number of proteins that detected α-helical propensities in conformational ensembles correspond well to experimentally determined α-PresMos. There are also mispredictions listed in Table 1 (*HBV, HMGA, and* TMOD) that may result from inherent limitations of force fields developed for structured proteins. Implementing a force field in DMD for disordered protein as have been recently done for the Amber force field [28] could enhance the prediction accuracy of our approach. Although RX-DMD describes an ensemble of conformations, which is the best proxy to describing disorder proteins, it is challenging to correlate computational ensembles with experimental data because of limited experimental structural information on IDPs and the type of data to be correlated (e.g. the average of interatomic distances in an ensemble can be reproduced even if their distribution is completely different from the reference; vice versa the same distributions do not necessarily reflect the same average interatomic distances [8]; Figure S3 in File S1). Moreover, the time averaging in NMR experiments is more pronounced, thus helices forming and breaking up slowly can provide signals similar to that of helices with fast kinetics (fast transitions between structured and unstructured states). In simulations, the time resolution of the motion is much higher. Nevertheless, the accuracy of RX-DMD in predicting α-PreSMos suggests that DMD captures important physicochemical features of disordered proteins and is a valid *ab initio* method to study their structural features. Its combined application with other knowledge-based methods (e.g. ELM, ANCHOR) may reduce the false positive rate of these latter. Our results also suggest that RX-DMD can provide insights into the mechanism of binding of disorder proteins (Table S1 in File S1), and it may be used as a high performance tool to investigate structural and dynamic properties of IDPs in delineating their function. To make the method easily accessible for researchers without any computational background, a web server is being developed for predicting α-PreSMos via RX-DMD.

## Supporting Information

File S1 **Figure S1–S3 and Table S1.** Figure S1. α-helical propensities in KID using RX-DMD. RX-DMD simulations using 8 replicas were performed for all the experimentally investigated proteins with PresMo, collected in the review of Lee et al. [1] α-helical propensities of the conformational ensembles at each simulation temperature were determined and plotted as described in Methods. KID is shown as an example, while the results for all other proteins and temperatures can be found at http://disorder.hegelab.org. Green boxes: PresMo regions determined experimentally [2], [3]. Figure S2. Comparing RX-DMD and Agadir predictions. Helical contents of the 24 protein segments with experimentally determined PreSMos were also predicted using Agadir [4]. From 65 PreSMos in these proteins 18 were detected by Agadir at 5% threshold based on the work of Bystroff and Garde [5], while 45 were detected by RX-DMD. Interestingly, in many cases when Agadir and RX-DMD match exactly the same regions, such as in the case of the first PreSMo in KID presented in this figure. In addition, RX-DMD finds 32 PreSMos not detected experimentally, while Agadir does 8. It is important to note that these numbers do not necessary indicate false positives, since many PreSMos might exist and be not visible by NMR because of their rate of conformational transitions. Agadir and RX-DMD predictions are identical in many cases also in the case of PresMos not detected by experiments that may be employed to filter out false positives. Black: RX-DMD; blue: Agadir. Figure S3. Rg distribution of α-Synuclein ensembles from RX-DMD simulations is similar to that determined in experiments [6]. Table S1. Prediction of RX-DMD compared to complexes observed in X-ray structures. Four segments from transmembrane proteins and 86 segments out of 97 PDB entries, which exhibited at least one region with 20% of helical propensity, are listed. From this 90 segments in 36 cases were the prediction in good agreement with the experimental results (only a few a.a. difference). This observation suggests that significant portion of PresMos have a direct role in binding and are MoREs. *sequences and their boundaries can be found at http://disorder.hegelab.org. **marks selected membrane proteins.
(PDF)
