# Supplementary material for: Discrete Molecular Dynamics Can Predict Helical Prestructured Motifs in Disordered Proteins
Source: PLoS One. 2014 Apr 24;9(4):e95795. doi: 10.1371/journal.pone.0095795 (PMC3998973; doi:10.1371/journal.pone.0095795)
Supplement: File S1 — Figure S1–S3 and Table S1. Figure S1. α-helical propensities in KID using RX-DMD. RX-DMD simulations using 8 replicas were performed for all the experimentally investigated proteins with PresMo, collected in the review of Lee et al. [1] α-helical propensities of the conformational ensembles at each simulation temperature were determined and plotted as described in Methods. KID is shown as an example, while the results for all other proteins and temperatures can be found at http://disorder.hegelab.org. Green boxes: PresMo regions determined experimentally [2], [3]. Figure S2. Comparing RX-DMD and Agadir predictions. Helical contents of the 24 protein segments with experimentally determined PreSMos were also predicted using Agadir [4]. From 65 PreSMos in these proteins 18 were detected by Agadir at 5% threshold based on the work of Bystroff and Garde [5], while 45 were detected by RX-DMD. Interestingly, in many cases when Agadir and RX-DMD match exactly the same regions, such as in the case of the first PreSMo in KID presented in this figure. In addition, RX-DMD finds 32 PreSMos not detected experimentally, while Agadir does 8. It is important to note that these numbers do not necessary indicate false positives, since many PreSMos might exist and be not visible by NMR because of their rate of conformational transitions. Agadir and RX-DMD predictions are identical in many cases also in the case of PresMos not detected by experiments that may be employed to filter out false positives. Black: RX-DMD; blue: Agadir. Figure S3. Rg distribution of α-Synuclein ensembles from RX-DMD simulations is similar to that determined in experiments [6]. Table S1. Prediction of RX-DMD compared to complexes observed in X-ray structures. Four segments from transmembrane proteins and 86 segments out of 97 PDB entries, which exhibited at least one region with 20% of helical propensity, are listed. From this 90 segments in 36 cases were the prediction in good agreement with the experimental r [file pone.0095795.s001.pdf]

SUPPORTING INFORMATION

Discrete molecular dynamics can predict prestructured motifs in disordered proteins

Dániel Szöllősi, Tamás Horváth, Kyou-Hoon Han, Nikolay V. Dokholyan, Péter Tompa, Lajos Kalmár, Tamás Hegedűs

MTA-SE Molecular Biophysics Research Group, Hungarian Academy of Sciences, Budapest, Hungary

Figure S1 α-helical propensities in KID using RX-DMD. RX-DMD simulations using 8 replicas were performed for all the experimentally investigated proteins with PresMo, collected in the review of Lee *et al.[1]* α-helical propensities of the conformational ensembles at each simulation temperature were determined and plotted as described in Methods. KID is shown as an example, while the results for all other proteins and temperatures can be found at http://disorder.hegelab.org. Green boxes: PresMo regions determined experimentally [2,3].

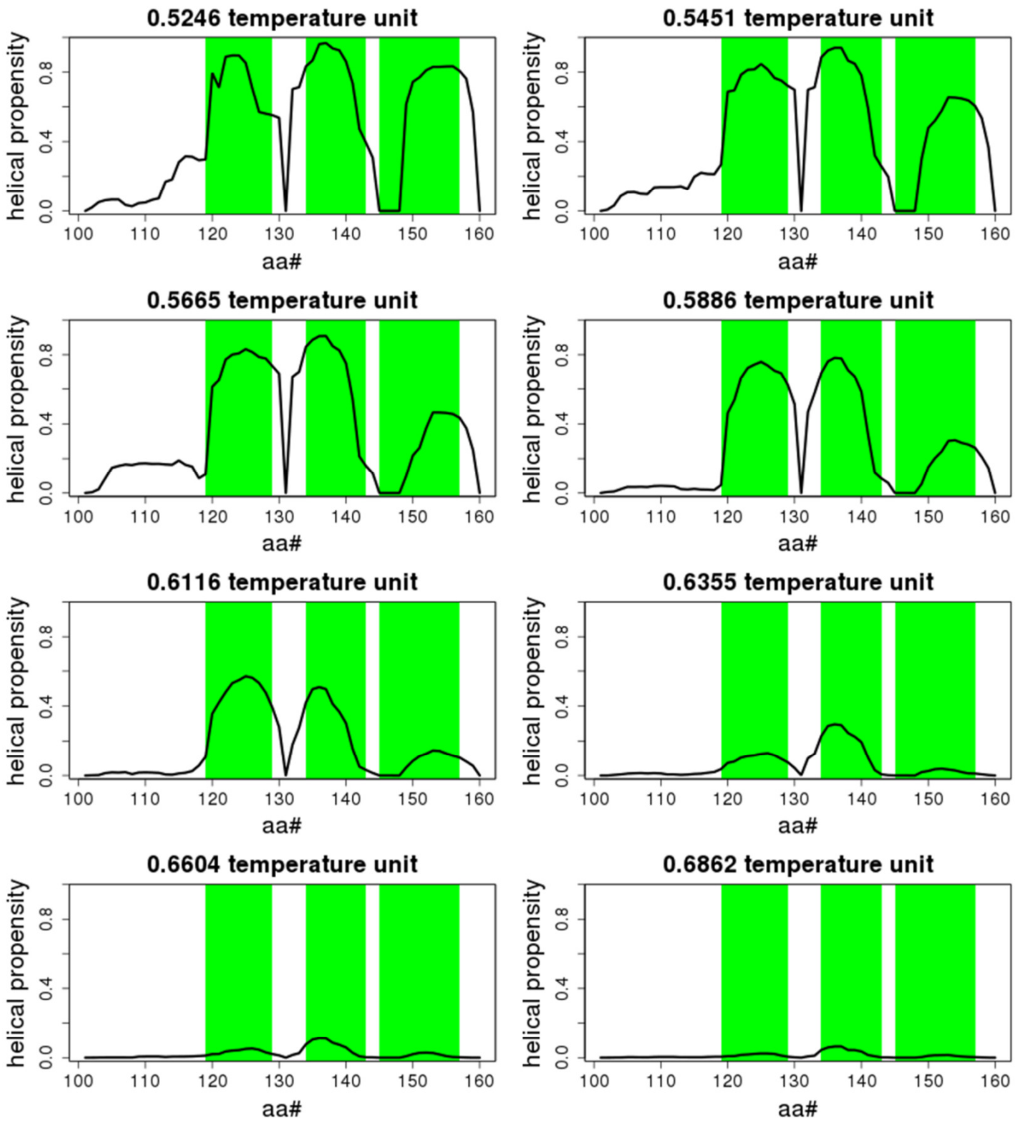

Figure S2 Comparing RX-DMD and Agadir predictions. Helical contents of the 24 protein segments with experimentally determined PreSMos were also predicted using Agadir [4]. From 65 PreSMos in these proteins 18 were detected by Agadir at 5% threshold based on the work of Bystroff and Garde [5], while 45 were detected by RX-DMD. Interestingly, in many cases when Agadir and RX-DMD match exactly the same regions, such as in the case of the first PreSMo in KID presented in this figure. In addition, RX-DMD finds 32 PreSMos not detected experimentally, while Agadir does 8. It is important to note that these numbers do not necessary indicate false positives, since many PreSMos might exist and be not visible by NMR because of their rate of conformational transitions. Agadir and RX-DMD predictions are identical in many cases also in the case of PresMos not detected by experiments that may be employed to filter out false positives. Black: RX-DMD; blue: Agadir.

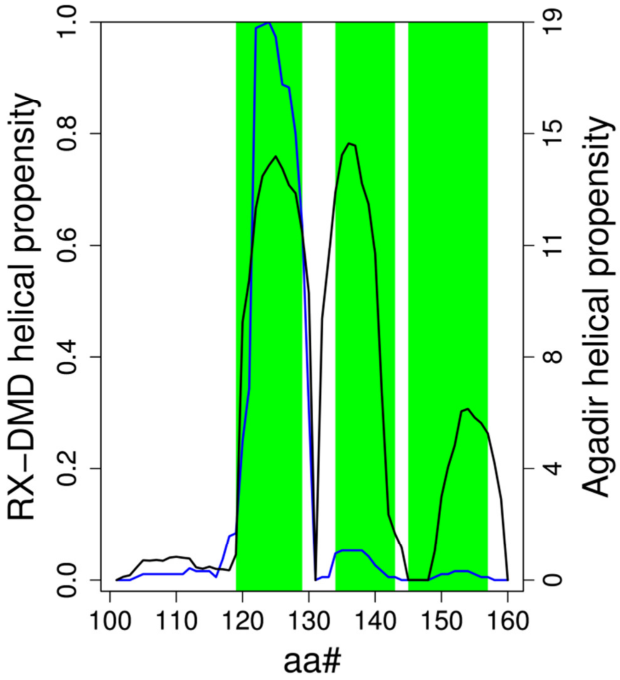

Figure S3 R_g distribution of α-Synuclein ensembles from RX-DMD simulations is similar to that determined in experiments [6].

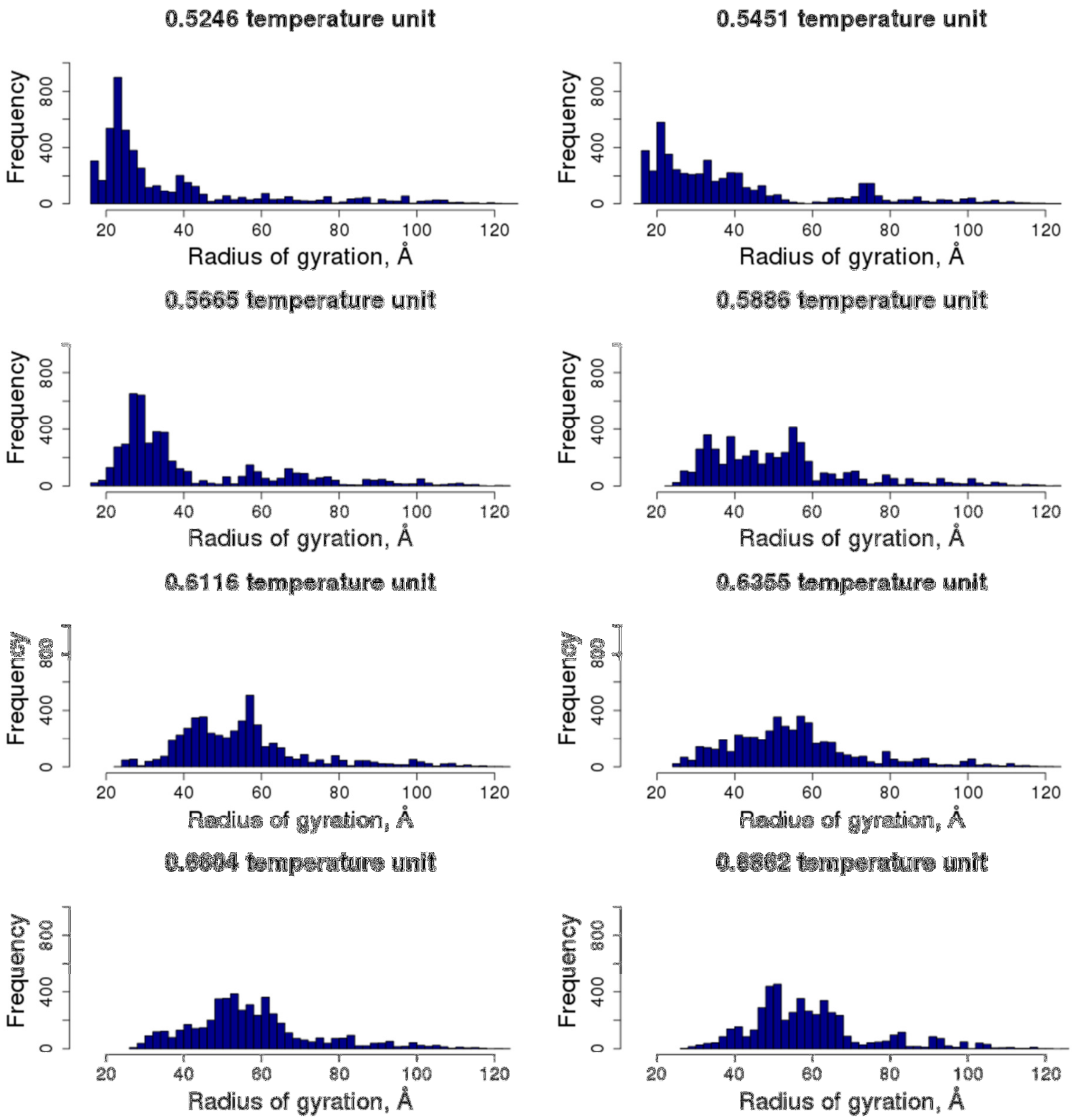

Table S1. Prediction of RX-DMD compared to complexes observed in X-ray structures. Four segments from transmembrane proteins and 86 segments out of 97 PDB entries, which exhibited at least one region with 20% of helical propensity, are listed. From this 90 segments in 36 cases were the prediction in good agreement with the experimental results (only a few a.a. difference). This observation suggests that significant portion of PresMos have a direct role in binding and are MoREs.

*sequences and their boundaries can be found at http://disorder.hegelab.org

**marks selected membrane proteins

UniProtACC(PDB ID)	Experimental MoREs	RX-DMDPresMos*
Q13936 (1T0J)**	428-445	408-444, 491-521
Q13936 (2BE6)**	1660-1673	1631-1651, 1662-1685, 1694-1700
P19634 (2BEC)**	503-545, 622-689	503-552, 554-562, 627-636, 658-683
Q9UKV5 (3H8K)**	574-601, 622-640	573-603,622-641
A1L0Z0 (3AFR)	624-634	628-634,639-644
B2RA72 (3EAB)	171-191	177-195
O00401 (2VCP)	433-451	434-443
O00512 (2VP7)	348-373	352-373
O00512 (2VP7)	175-201	175-180,182-194,201-209
O15047 (4EWR)	1490-1499	1502-1513
O15234 (3EX7)	216-246	205-226,230-240
O33246 (3M91)	25-51	37-61
O35274 (3EGG)	446-486	464-469,497-502
O43312 (2D1K)	725-753	726-737
O43516 (2A41)	29-60	34-36
O88797 (3H8D)	676-713	680-689,694-710
O96013 (2OV2)	10-44	14-31,38-42
P01094 (1DP5)	2-32	5-18,25-37
P01096 (1OHH)	29-63	11-21,55-77
P03255 (2R7G)	40-49	22-30,41-50
P03255 (2R7G)	53-85	41-50
P04637 (2L14)	372-388	367-388
P04637 (2L14)	14-54	16-25,40-41,47-54
P05412 (1A02)	272-308	256-290,293-312
P05889 (1FGL)	218-228	203-244
P09052 (2IHS)	184-203	174-184,195-200
P0AFX7 (3M4W)	169-190	163-194
P0DJ89 (2LNH)	268-307	269-282,316-326
P13199 (2YKA)	107-120	109-116
P14598 (1K4U)	359-387	372-376,380-388
P14653 (1B72)	177-185	175-186

P17676 (1H88)	292-334	274-281,289-323,325-333
P23615 (3OAK)	239-263	240-250,258-269
P25054 (1JPP)	1021-1034	1003-1015,1024-1038
P25054 (1JPP)	1468-1496	1486-1493
P26368 (1JMT)	90-111	83-91,104-113
P27321 (3BOW)	571-588	565-583
P27782 (3OUW)	48-60	31-35,53-54
P35465 (1E0A)	75-108	100-107
P35579 (3ZWH)	1893-1935	1874-1904,1907-1923
P36108 (2V6X)	184-206	167-175
P38968 (2QTV)	907-942	913-920
P40019 (2JSS)	65-125	70-86,88-122
P40056 (3ZS9)	6-38	3-22
P40344 (3T7G)	133-142	129-145,152-155
P40855 (3AJB)	15-34	9-31
P42226 (1OJ5)	795-808	800-810
P42768 (1CEE)	230-277	260-274,287-290
P45481 (1KBH)	2060-2117	2044-2046,2065-2076,2099-2110
P46937 (3KYS)	52-73	65-71
P49054 (2DOI)	90-108	72-127
P52292 (1QGK)	11-54	10-23,25-52
P55036 (1P9D)	271-298	284-302
P68807 (2DF4)	3-100	9-19,30-41,52-69,74-87
P70211 (3PZD)	1410-1443	1418-1443
P70365 (4DMA)	691-701	687-700
Q03164 (3U88)	107-135	127-132,145-147
Q03164 (3U88)	3755-3770	3739-3747,3767-3784
Q04839 (3LCN)	126-148	123-138,144-163
Q12420 (3PLV)	37-56	25-30,59-60
Q12420 (3PLV)	7-24	9-19,26-36
Q15648 (1RJK)	640-650	643-651
Q5U5Q9 (3A1Q)	81-120	69-98,102-124
Q5VZK9 (3LK3)	971-1004	959-968,977-987
Q61026 (3MNE)	741-750	738-751,765-768
Q6R3M4 (2KWU)	680-709	695-708
Q7DB77 (2ZQK)	277-314	277-282,284-287,311-318,320-321
Q87GE5 (3M1F)	130-151	133-144
Q8BN74 (2QGT)	687-698	681-696
Q8WXE9 (2JXC)	308-340	330-344
Q92934 (1G5J)	103-127	102-127

Q95PA1 (1L4A)	51-75	34-80
Q96CF2 (3C3R)	221-233	209-232
Q99ML1 (2ROC)	131-156	127-162,167-175
Q9BY43 (3C3O)	210-222	206-220
Q9H444 (3C3Q)	209-224	204-221
Q9HAU5 (2WJV)	1105-1128	1107-1128
Q9KWH9 (1F02)	294-317	285-289,316-333
Q9U1K1 (3UE5)	463-481	464-475
Q9U1K1 (3UE5)	2-19	28-37
Q9UBK2 (3CS8)	141-152	141-148
Q9UBK2 (3CS8)	205-218	207-215
Q9Y3E7 (2XZE)	202-222	196-220
Q9Y5B0 (1J2X)	944-961	943-960
Q9Y5X1 (3LGE)	164-182	173-186
Q9Y618 (2GPV)	1319-1330	1316-1332
Q9Y618 (2GPV)	2337-2345	2353-2364
Q9Y6J0 (1N6J)	2159-2184	2166-2189
Q9Y6Q9 (3L3Z)	735-746	729-744,749-754
Q9YGY0 (1QZ7)	467-482	461-464,470-480

References

1. Lee SH, Kim DH, Han JJ, Cha EJ, Lim JE, et al. (2012) Understanding pre-structured motifs (PreSMos) in intrinsically unfolded proteins. Curr Protein Pept Sci 13: 34-54.
2. Radhakrishnan I, Perez-Alvarado GC, Dyson HJ, Wright PE (1998) Conformational preferences in the Ser133-phosphorylated and non-phosphorylated forms of the kinase inducible transactivation domain of CREB. FEBS Lett 430: 317-322.
3. Radhakrishnan I, Perez-Alvarado GC, Parker D, Dyson HJ, Montminy MR, et al. (1997) Solution structure of the KIX domain of CBP bound to the transactivation domain of CREB: a model for activator:coactivator interactions. Cell 91: 741-752.
4. Lacroix E, Viguera AR, Serrano L (1998) Elucidating the folding problem of alpha-helices: local motifs, long-range electrostatics, ionic-strength dependence and prediction of NMR parameters. J Mol Biol 284: 173-191.
5. Bystroff C, Garde S (2003) Helix propensities of short peptides: molecular dynamics versus bioinformatics. Proteins 50: 552-562.
6. Allison JR, Varnai P, Dobson CM, Vendruscolo M (2009) Determination of the free energy landscape of alpha-synuclein using spin label nuclear magnetic resonance measurements. J Am Chem Soc 131: 18314-18326.
